# Occipital condyle fracture and lower cranial nerve palsy after blunt head trauma – a literature review and case report

**DOI:** 10.1186/s13032-015-0024-3

**Published:** 2015-04-11

**Authors:** Nils Christian Utheim, Roger Josefsen, Per Hjalmar Nakstad, Torfinn Solgaard, Olav Roise

**Affiliations:** Department of Neurosurgery, Division of Surgery and Neuroscience, Oslo University Hospital, Oslo, Norway; Department of Neuroradiology, Division of Diagnostics and Intervention, Oslo University Hospital, Oslo, Norway; Department of Orthopedics, Division of Surgery and Neuroscience, Oslo University Hospital, Oslo, Norway; Institute of Clinical Medicine, Faculty of Medicine, University of Oslo, Oslo, Norway

**Keywords:** Cranial nerve palsy, Occipital condyle fracture, Collet-Sicard-Syndrome

## Abstract

**Background:**

Lower cranial nerve (IX-XII) palsy is a rare condition with numerous causes, usually non-traumatic. In the literature it has been described only a few times after trauma, mostly accompanied by a fracture of the occipital condyle. Although these types of fractures have rarely been reported one could suspect they have been under-diagnosed. During the past decade they have been seen more frequently, most probably due to increased use of CT- and MRI-scanning. The purpose of this review is to increase the awareness of complications following injuries in the craniocervical region.

**Methods:**

We based this article on a retrospective review of the medical record of a 24-year old woman admitted to our trauma center after being involved in a car accident and a review of the literature on occipital condyle fractures associated with lower cranial nerve palsy.

**Results:**

The multitraumatized patient had suffered a dislocated occipital condyle fracture. Months later she was diagnosed with palsy to cranial nerve IX-XII. Literature review shows that occipital condyle fractures are rare as isolated injuries and are in many cases accompanied by further injuries to the cervical spine and soft tissue structures, in many cases ending with severe disability. The exact mechanism leading to these injuries cannot always be explained.

**Conclusion:**

Recognition of soft tissue injuries in patients with blunt head trauma is important. CT findings involving the craniocervical junction in these patients advocates further investigations including a thorough neurological examination and liberal use of MRI.

## Introduction

Cranial nerve palsy involving the four lower cranial nerves (IX-XII) is known as the Collet-Sicard Syndrome. Collet was the first to describe this in 1915 [1] as “glossolaryngoscapulopharyngeal” hemiplegia in a patient with a gunshot injury to the mastoid. Two years later, in 1917, Sicard described it as “the syndrome of the condyloposterior lacerated foramen” [2]. Clinical signs can be hoarseness and difficulties swallowing (CN IX & X), shoulder and neck weakness (CN XI). Furtheron palsy of CN XII indicate problems involving the tongue muscles which can lead to difficulties talking, chewing and swallowing. Depending on the cause symptoms can occur slowly over time or sudden (ie. after trauma). This can affect the prognosis regarding recovery which is being discussed below.

Lower cranial nerve palsy is a rare condition and its causes are numerous. It has amongst others been attributed with malignant skull base lesions such as multiple myeloma [3], matastasis of prostate cancer [4,5], internal carotid dissection [6,7], hypoglossal Schwannoma [8] and Jefferson fracture [9,10].

Furthermore lower cranial nerve palsy is attributed with occipital condyle fractures (OCF). Bell was the first to describe fractures of the occipital condyle in 1817 [11]. These are rarely reported in literature and it is unclear whether these fractures are rare or under-diagnosed. They have been seen more frequently during the past decade, most probably due to increased use of CT- and MRI-scanning [12,13]. They are rare as isolated injuries and are mostly accompanied by other injuries of the cervical spine. Reviewing the literature regarding OCF one study found that 22% of patients with OCF had associated injuries of the cervical spine, with the majority of injuries being fractures of C1 and C2 [14].

We report a case of a 24-year old woman with left-sided unilateral palsy of the four lower cranial nerves who had suffered a right sided occipital condyle fracture.

To the best of our knowledge this injury has rarely been reported before. We suggest that this injury with subtile clinical symptoms may be overlooked from time to time. We therefore focus on this entity with regard to development of lower cranial nerve palsy.

## Materials and methods

This article is based on a review of the literature on occipital condyle fractures associated with lower cranial nerve palsy, as well as a retrospective review of the medical record of a 24-year old woman admitted to our trauma center after being involved in a car accident. A written informed consent was obtained from the patient.

We performed a Medline search for occipital bone injury which yielded 352 citations from 1950-2011. This was combined with a search for Collet-Sicard-Syndrome as a keyword which yielded 46 citations. All abstracts of the 398 articles were read searching for lower cranial nerve palsy combined with occipital condyle fracture. We found 22 articles referring to occipital condyle fractures with lower cranial nerve palsy.

### Case report

**Figure 1:**
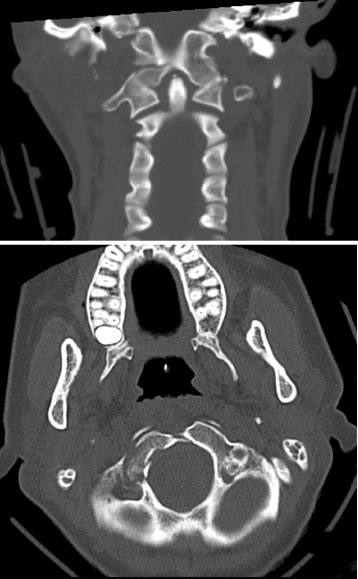
Comminute fracture through the occipital condyle on the right side in coronal and axial views.

Glasgow Coma Score (GCS) at the scene of accident directly after the trauma was reportedly seven with a normal pupillary response. Prior to transportation to the trauma center the patient was intubated and a cervical collar was applied. She was polytraumatized with multiple injuries; pneumothorax, pulmonary contusions, fractures of the upper and lower extremities. The cranial CT-scan revealed a dislocated fracture of the right occipital condyle (Figure 1) and a small amount of blood in the interpeduncular cistern.

She underwent surgery for the injuries of the extremities. Due to the fracture of the occipital condyle her neck was immobilized with a hard collar for 12 weeks. She remained hospitalized in the intensive care unit (ICU) for six weeks due to her severe injuries including a brain edema, diagnosed with CT. The brain edema was believed to be the most probable cause for her low initial GCS-score of seven.

**Figure 2:**
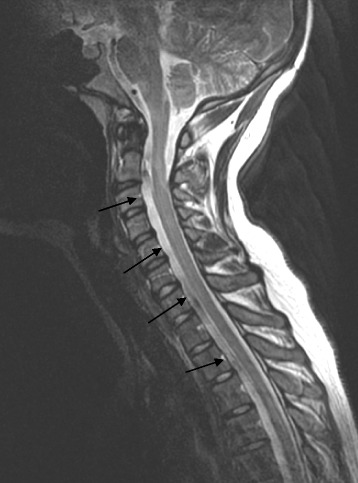
T2-weighted MRI of the spinal canal in the sagittal plane demonstrates a probable arachnoid cyst located anterior to the spinal cord from C2 and downwards. Additional MRI of the thoracic spine showed extension down to Th10. The MRI was performed one year after the accident.

**Figure 3:**
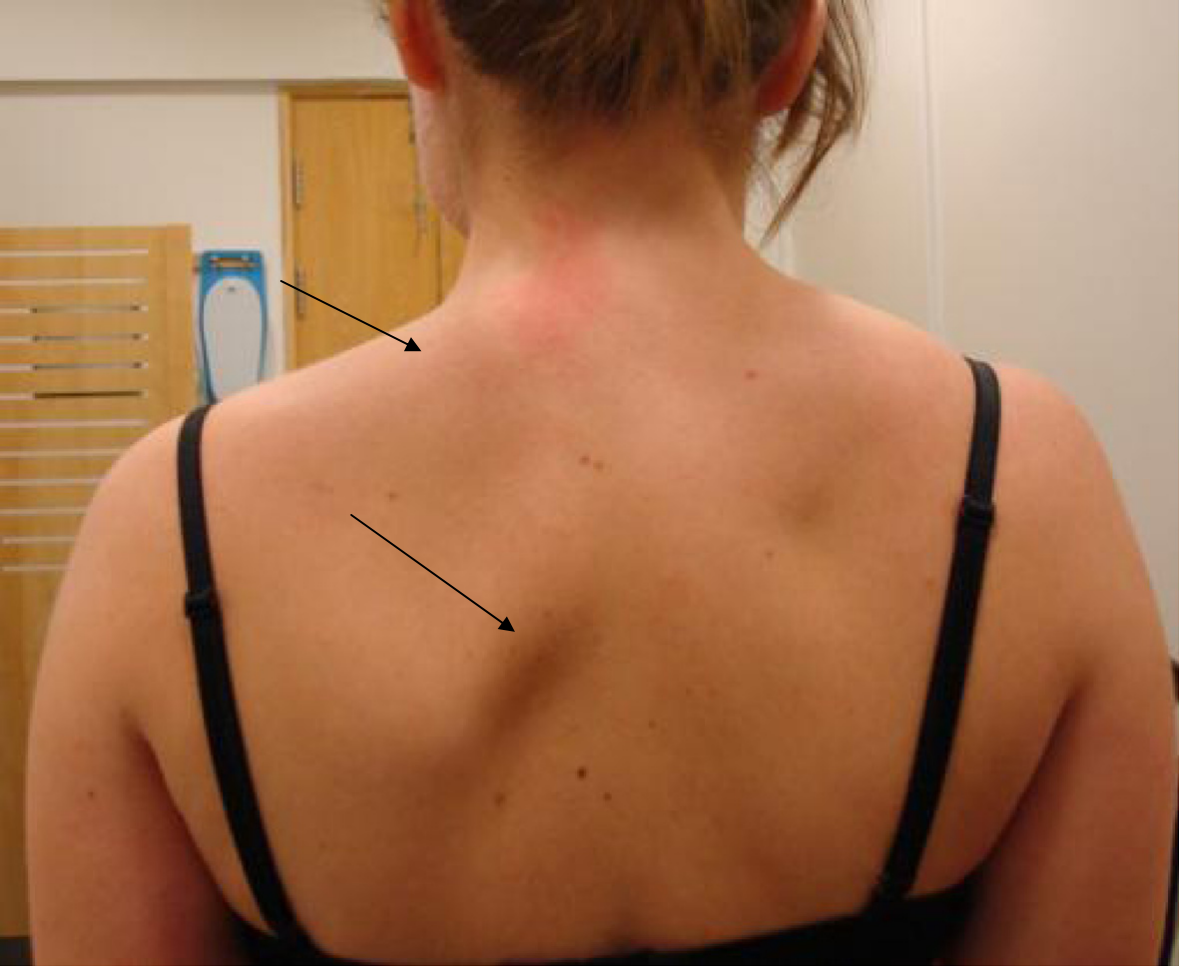
Left sided atrophy of the trapezius muscle and scapular winging (arrows).

According to the the patient she started noticing hoarseness and difficulties swallowing about six weeks after the accident. At this point she was gradually recovering from sedation and assisted breathing. After hospitalization she was admitted to the rehabilitation-unit. Due to lack of progression and weakness in her left shoulder, despite training, she was referred to an MRI-scan of the neck and thoracal region. MRI was not conclusive in terms of soft tissue injury. On the other hand it did reveal a cystic lesion anterior to the medulla with extention from C2 to Th10 (Figure 2). The lesion is most likely to be intradural and probably represents the accidental finding of an arachnoid cyst. Later neurography showed axonal injury to the left accessory nerve (XI). Three years after the accident the patient still suffers from paresis of the left trapezius and sternocleidomastoid muscle and insignificant paresis of the throat muscles. She is partly disabled due to pain in the neck and the left shoulder where she has a scapular winging (Figure 3).

Our patient has all the expected symptoms of the Collet- Sicard-syndrome except affection of the tongue muscles (CN XII). Injury to just two or three of the lower cranial nerves is reported in several of the reviewed cases and is referred in more detail below. Thus one has a good basis for the diagnosis of the syndrome in this trauma patient.

## Discussion

The dural sheet reaching from C2-Th10 is most likely and arachnoid cyst, however one cannot rule out the possibility of an epidural hematoma. However the combination of lower cranial nerve palsy accompanied by occipital condyle fractures and spinal epidural hematoma only yielded two citations [15,16] in a Medline-search.

The finding of a cystic lesion extending from C2-Th10 needs to be discussed whether it represents an epidural hematoma or not. The clinical symptoms were cranial nerve palsy of CN IX, X and XI. She had no paresis to her upper or lower limbs and no affection of bladder and sphincter function. With the extent and size of the lesion one would expect some affection of the medulla, which is not the case. The cystic lesion has been scanned with MRI with an interval of three years and there is no change of size and signal in that time. High signal in T2-weighted images on both scans indicate that this is fluid and not a hematoma. The unchanged finding of septal layers also support that the finding most likely is an arachnoid cyst and that this lesion was not caused by the trauma. We therefore strongly believe this is an accidental finding in our patient.

Occipital condyle fractures are associated with high-energy blunt trauma with significant cranio-cervical torque or axial loading. In most cases neurological deficits more often seem to be related to the severity of the head and neck injury than to the occipital condyle fracture itself. Many patients who suffer an occipital condyle fracture are multi-traumatized and do not survive the initial trauma [17].

The clinical symptoms and signs of patients after a blunt head trauma may be varying and diverse. Our patient developed paralysis of the sternocleidomastoid and trapezius muscles and hoarseness in addition to difficulties swallowing. The latter is indicating palsy of cranial nerve IX, X and XI. The possible causes of these deficits are numerous. The most important findings in our case report were a dislocated occipital condyle fracture and suspected soft tissue injury which is discussed in more detail below.

### Anatomy and biomechanics of the craniocervical junction (CVJ)

Knowledge of the anatomy and function of the structures in the posterior fossa and the close relation of the occipital condyles to the brain-stem and the structures passing through the foramen magnum is of great importance for understanding the nature of these injuries.

The craniocervical joint is a complex joint including the atlas, dens of axis and the occipital condyles [18]. Numerous ligamentous structures provide stability for complex movements of the joint. These allow rotation, flexion and extension of the joint without risking injury to the neural structures passing through.

The occipital condyles form the lateral parts of the foramen magnum. They are perforated by the hypoglossal canals which contain the hypoglossal nerves (cranial nerve XII) that provide the motor innervation of the tongue-muscles. Directly lateral to the occipital condyles are the jugular foramina, containing the jugular vein and cranial nerve IX, X, XI. These nerves provide the innervation of the throat, sternocleidomastoid and trapezius muscle.

The atlas consists of a posterior and an anterior arch lacking a vertebral body. Furthermore it provides two lateral masses which articulate with the occipital condyles of the skull and form the atlantooccipital joint. The space of the usual vertebral body is occupied by the odontoid process of the axis to allow for a greater amount of rotation in the superior (upper) part of the neck. The odontoid process articulates with the arch of the atlas ventrally and a ligamentous structure (transverse ligament) dorsally.

The bony parts of these four joints altogether provide poor stability. The most important structures providing ligamentous stability to the craniocervical junction are the alar ligaments, the cruciate ligament and the tectorial membrane [19]. The alar ligaments reach from the occipital condyles to the tip of the odontoid process and limit rotation, whilst the tectorial membrane and cruciform ligament run anteriorly to the medulla from the dorsal part of the odontoid to the ventral part of the foramen magnum and limit hyperextension.

The alar ligaments are restraining the craniocervical rotation and lateral bending. Consequently the mechanism of injury is either rotation, lateral bending or the combination of both. Injury of the alar-ligament may lead to dislocation of the fractured occipital condyle. The combination of fracture of the condyle and additional injury to the alar ligament is therefore considered to be unstable by many authors.

### Classification

Regarding occipital condyle fractures, Anderson and Montesano [20] published a classification guideline in 1988 based on a retrospective review of 6 patients who were scanned with either CT or conventional tomography. These guidelines were in 1997 modified by Tuli et al. [21] determining instability according to CT and MRI-scans considering the extent of ligamentous injury and occiput-C1-C2 rotation and translation.

**Figure 4:**
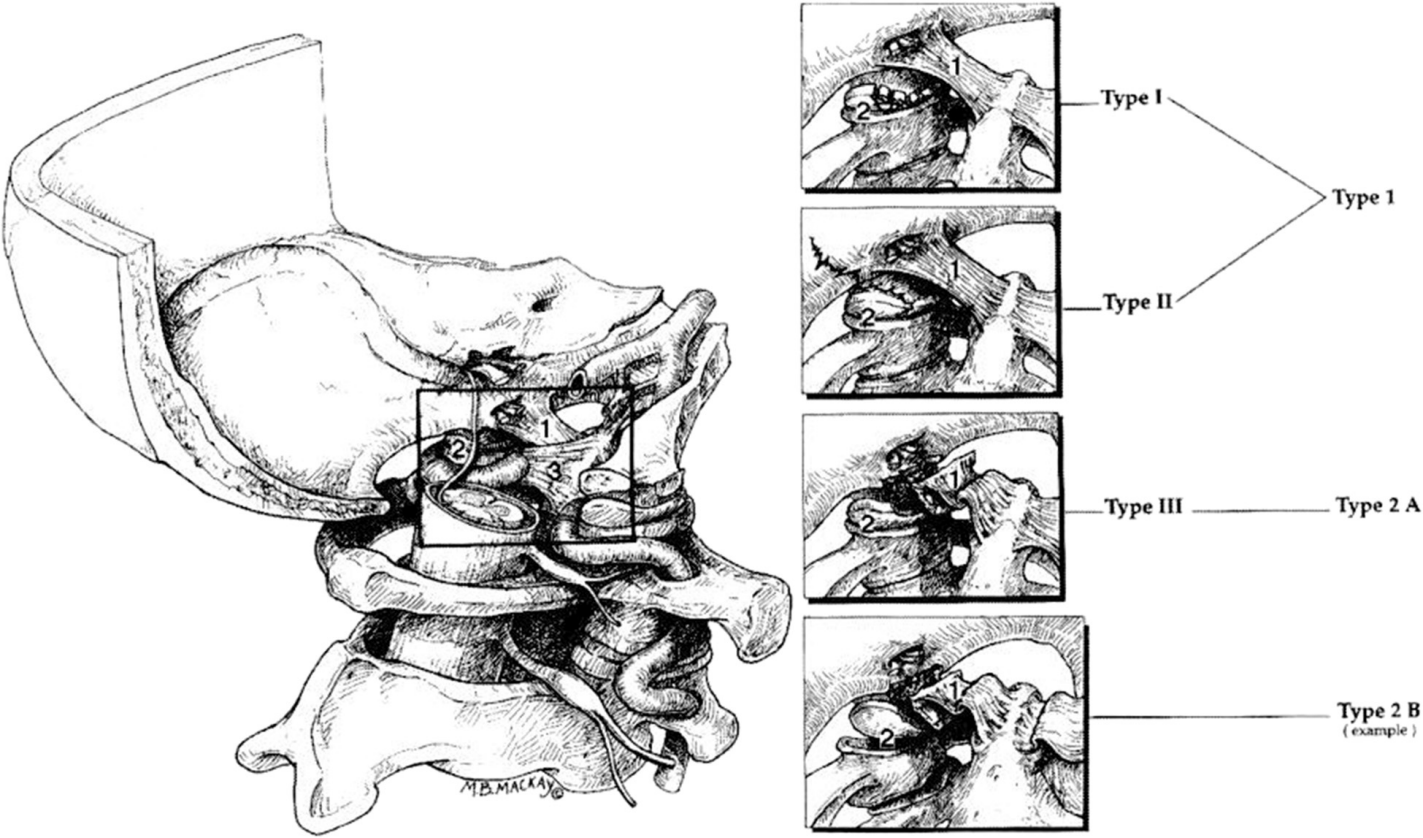
Types of OCF based on the Anderson and Montesano classification system (Types I-III) compared with the Tuli classification system (Types 1, 2A, and 2B), it shows the left craniocervical junction from its medial aspect. The dura and the inferior aspect of the alar ligament have been removed to show the fractured condyles in the fracture types. Tuli S, Tator C.H, Fehlings M.G, Mackay M (1997) Occipital condyle fractures. Neurosurgery;41:368-76.

Occipital condyle fractures are by Tuli et al. divided into three types [21] based on dislocation, rotation and translation of the 0-C1-C2-complex, and ligamentous injury verified by MRI (Figure 4). Type 1 is an undisplaced fracture. Type 2A is seen as stable if no findings of ligamentous injury is demonstrated on MRI, without rotation or translation in the 0-C1-C2 complex. Type 2B fractures are potentially unstable injuries according to Tuli et al. It is a displaced fracture of the occipital condyle with MRI evidence for ligamentous disruption, or rotation and translation in the CT-scan.

**Figure 5:**
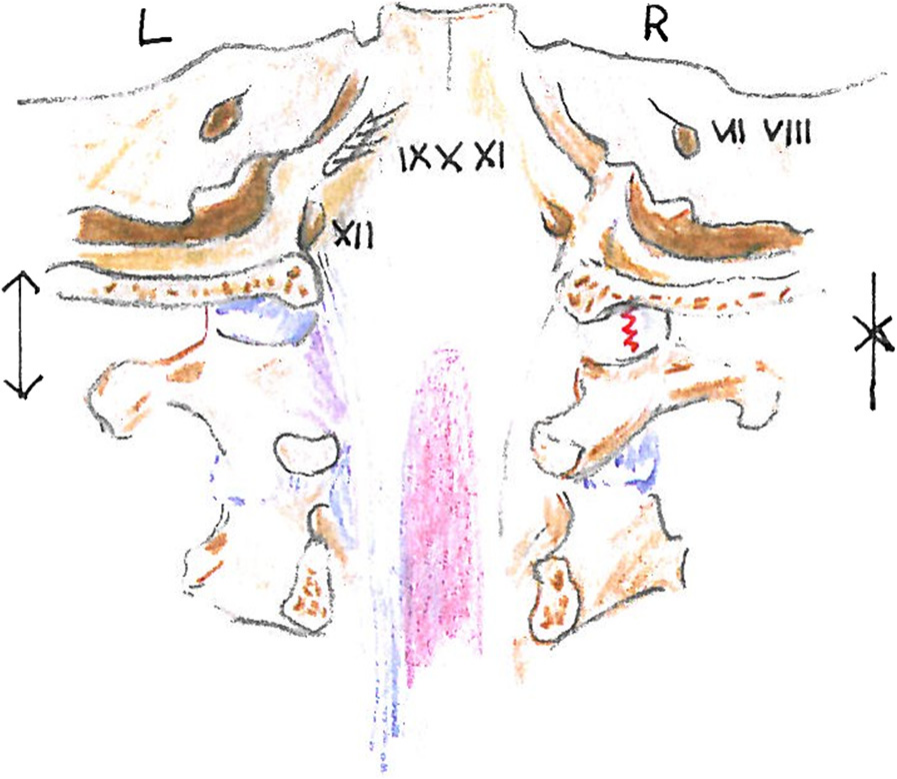
Demonstrates the suggested injury mechanism; compression (arrow) on the right side causes the occipital condyle fracture and a simultaneous stretching (arrow) on the contralateral side leads to injury of the left sided nerves. Dorsal view. (after Frank H. Netter: Atlas of Human Anatomy 4th ed., plate 11,22).

Figure 1 shows the fractured right occipital condyle with a dislocated fragment into the foramen magnum. Later MRI showed no sign of ligamentous instability. This indicates a type 2A fracture which is considered stable. Follow-up CT showed no sign of further dislocation of the fragment. The MRI was not performed in the first few weeks after inury and one must always take into cencern that the edema will not show in the later phases of the injury. The fact that the dislocated fragment is in close relation to cranial nerve IX, X, XI indicates that this injury is the most probable cause of the patient’s cranial nerve palsy. The exact mechanism is still unclear since the palsy is left-sided and the fracture is on the contralateral side. Numerous possible mechanisms exist including nerve stretching (Figure 5), nerve rootlet avulsion and compression.

Two other reports suggested that the formation of scar tissue and callus, which cause compression of the nerve in the posterior fossa after OCF could be the cause of cranial nerve XII palsy [22,23]. In our case the patient started noticing the symptoms waking up in ICU six weeks after trauma. Most likely the palsy to cranial nerve IX, X, XI was already present directly after the accident with the patient not able to notice as she was intubated and sedated. Castling et al [22] and Orbay et al. [23] reported cranial nerve palsy six days and two months after the initial trauma. Earlier reports indicate that immediate deficits have a lower recovery-rate than secondary palsies [17]. Our patient most probably suffered a primary palsy and this is most likely to be permanent. In an MRI-scan four years after the initial injury the dural sheat duplication reaching from C2-Th10 was still present. The occipital condyles and the dislocated fragment seemed unchanged.

A spinal epidural hematoma can result in serious neurological deficits and they are reported to be present in 0.5-7.5% of all vertebral fractures [24]. As mentioned above occipital condyle fractures with associated epidural hematomas have only been reported in very few cases. As for our case the dural sheet duplication is not a hematoma and just an incidental finding of an arachnoid cyst and is not likely to cause palsy to the lower cranial nerves. If this was to represent a hematoma one would due to the size and extent of the finding (Figure 2) expect the patient to have additional neurological findings. Furthermore one would expect symptoms indicating myelopathy like hyperreflexia, spastic paresis or loss of bladder and anal sphincter control, indicating the medulla and first motoneuron being affected. In our case the patient had clinical findings with scapular winging (Figure 3) indicating peripheral nerve affection like paresis and muscular atrophy. Scapular winging is normally caused by dysfunction of either the trapezius or serratus anterior muscle. These muscles are innervated by the accessory (XI) and long thoracic nerve (Cervical branches 5-7) respectively. Neurography findings indicated damage to the accessory nerve with axonal injury and denervation. The long thoracic nerve was not investigated.

### Imaging

Fractures of the occipital condyles are in most cases confirmed with a CT scan following trauma to the head and neck. They have been more frequently diagnosed over the last 20 years, most certainly due to increased use of CT-scans in trauma patients.

### Treatment

Regarding treatment [25] of OCF, all three types of OCF have been treated either with immobilization with a stiff neck collar or a halo jacket or with no treatment at all. There are reports of three patients [14] with potentially unstable fractures who were operated to remove the dislocated fragment. In these cases all patients improved regarding cranial nerve palsy. It is however still uncertain if the surgery was responsible for the recovery of nerve function.

To date no study has produced sufficient evidence showing that patients treated with a hard neck collar have a better outcome than patients who do not receive any treatment, however the number of patients is small. Most trauma centers recommend treatment of occipital condyle fractures with a hard neck collar or a halo-frame for 6-12 weeks. Some case reports and studies regarding occipital condyle fractures, indicate that patients treated with a hard neck collar have a better outcome concerning cranial nerve palsy than untreated patients, but the number of patients in these cases are small as well [14].

Tuli et al. [21] recommend that undisplaced fractures without ligamentous injury (type 1) are not immobilized whilst a type 2A should be treated with a hard collar. Type 2B should be treated with a halo-vest or by surgical fixation.

This case is one of very few reported due to occipital condyle fracture and cranial nerve palsy. The dural sheat duplication is most probably a secondary finding. It is also unlikely that the dislocated fragment itself caused the palsy, hence the symptoms being left sided and the fracture of the occipital condyle being contralateral. We suggest that axonal injury due to stretching or nerve root avulsion is the most likely cause with a probable onset of the palsy directly after the accident (Figure 5). Although compression and the formation of scar tissue in relation to the nerves cannot be ruled out.

Fractures of the occipital condyles often indicate associated injuries of the cervical spine [14] and this demonstrates that fractures of the atlantooccipital joint might be associated with severe disabling injuries. Being aware of the delicate anatomy of the posterior fossa and of the close relation of cranial nerves and brain-stem to bony structures, severe disabling soft-tissue injuries can occur.

In conclusion, palsy of the lower cranial nerves after trauma is extremely rare. Our patient suffered both an occipital condylar fracture and contralateral cranial nerve palsy. Collated with the finding of an arachnoid cyst it makes our findings somewhat difficult to interprete. The Collet-Sicard-Syndrome following fractures of the occipital condyles is not often reported in literature, but should probably be more often considered in complex traumas of the craniocervical region.

CT and MRI of the craniocervical junction is therefore important in the clinical work-up of patients with blunt head and neck trauma.
